# Application of Iron Nanoparticles Synthesized from a Bioflocculant Produced by Yeast Strain *Pichia kudriavzevii* Obtained from Kombucha Tea SCOBY in the Treatment of Wastewater

**DOI:** 10.3390/ijms241914731

**Published:** 2023-09-29

**Authors:** Phakamani H. Tsilo, Albertus K. Basson, Zuzingcebo G. Ntombela, Nkosinathi G. Dlamini, Rajasekhar V. S. R. Pullabhotla

**Affiliations:** 1 Department of Biochemistry and Microbiology, Faculty of Science, Agriculture, and Engineering, University of Zululand, Private Bag X1001, Kwadlangezwa 3886, South Africa; phakamanitsilo90@gmail.com (P.H.T.); bassona@unizulu.ac.za (A.K.B.); ntombelaz@unizulu.ac.za (Z.G.N.); nathidlamini03@gmail.com (N.G.D.); 2 Department of Chemistry, Faculty Science, Agriculture, and Engineering, University of Zululand, Private Bag X1001, Kwadlangezwa 3886, South Africa

**Keywords:** iron nanoparticles, Kombucha tea SCOBY, removal efficiency, cytotoxicity, antimicrobial activity, bioflocculant

## Abstract

Studying the production of Iron (Fe) nanoparticles using natural substances is an intriguing area of research in nanotechnology, as these nanoparticles possess biocompatibility and natural stability, which make them useful for a variety of industrial applications. The study utilized Fe nanoparticles that were synthesized using a bioflocculant and applied to eliminate different kinds of pollutants and dyes found in wastewater and solutions. The study involved the generation of Fe nanoparticles through a bioflocculant obtained from *Pichia kudriavzevii*, which were evaluated for their flocculation and antimicrobial capabilities. The impact of the Fe nanoparticles on human embryonic kidney (HEK 293) cell lines was studied to assess their potential cytotoxicity effects. An array of spectroscopic and microscopic methods was employed to characterize the biosynthesized Fe nanoparticles, including SEM-EDX, FT-IR, TEM, XRD, UV-vis, and TGA. A highly efficient flocculating activity of 85% was achieved with 0.6 mg/mL dosage of Fe nanoparticles. The biosynthesized Fe nanoparticles demonstrated a noteworthy concentration-dependent cytotoxicity effect on HEK 293 cell lines with the highest concentration used resulting in 34% cell survival. The Fe nanoparticles exhibited strong antimicrobial properties against a variety of evaluated Gram-positive and Gram-negative microorganisms. The efficiency of removing dyes by the nanoparticles was found to be higher than 65% for the tested dyes, with the highest being 93% for safranine. The Fe nanoparticles demonstrated remarkable efficiency in removing various pollutants from wastewater. In comparison to traditional flocculants and the bioflocculant, biosynthesized Fe nanoparticles possess significant potential for eliminating both biological oxygen demand (BOD) and chemical oxygen demand (COD) from wastewater samples treated. Hence, the Fe nanoparticles synthesized in this way have the potential to substitute chemical flocculants in the treatment of wastewater.

## 1. Introduction

The availability of potable water is a major concern for the well-being of both humans and animals. The lack of water is linked to climate change, industrialization, and population expansion, especially in developing nations, in the majority of countries [1]. Various methods are employed in purifying water, including flotation and filtration, sedimentation, heating or boiling, as well as salting-in techniques. The utilization of chemicals for water purification has been regarded as the superior choice for treating water due to its high efficacy and cost-effectiveness [2]. Nevertheless, the usage of industrial substances has negative consequences due to their unfriendly nature, that is, their harmful effects on human well-being and the environment [3].

Conversely, the exceptional properties of bioflocculants have garnered attention in recent biotechnological and scientific research [4]. Microbes such as bacteria, algae, and fungi produce bioflocculants as by-products while they are growing, which are considered as secondary metabolites [5,6]. Bioflocculants are large molecules and include proteins, carbohydrates, glycoproteins, and nucleic acids that are produced from the growth of microorganisms, breakdown of cells, and substrates of metabolism. Bioflocculants possess distinctive features, such as being environmentally friendly, biodegradable, and not causing any secondary pollution. Bioflocculants have drawbacks such as expensive production, low yield, and lower efficiency compared to conventional flocculants [7]. Due to these reasons, bioflocculants are not considered commercially feasible.

Nanotechnology is hailed as the next major industrial revolution because it is viewed as the most rapidly advancing technology with the ability to create the foundation for merging technology and biotechnology [8]. This method has been applied in various academic disciplines and industries, including medicine, physics, chemistry, and biology [9,10]. Nanoparticles hold immense promise in multiple scientific domains owing to their superior biological and physicochemical characteristics in comparison to bulk substances [11]. Nanoparticles are substances that possess altered chemical and physical properties and fall within a size range of 1 to 100 nm [12]. Various techniques have been recorded for their production, encompassing both physical and chemical methods. Nonetheless, it appears that both methods have drawbacks, including the need for significant energy input and the use of hazardous reagents or substances. As a result of these limitations, biological approaches have been employed to create metallic nanoparticles and apply them in the treatment of wastewater [13]. Nanotechnology has been identified as viable solution for cost-effective water treatment, as it offers a promising approach that can deliver high-performance, highly efficient, flexible, and multi-functional processes [14]. The occurrence of antibiotic-resistant microorganisms in water poses a significant public health concern [15]. Furthermore, the biological method is commonly employed to monitor the desired size and shape of nanoparticles, which can have ecological benefits [16].

Recently, studies have investigated biosynthesis of iron nanoparticles [17,18]. This is due to the numerous distinctive properties of iron nanoparticles that render them valuable for a wide range of applications. These characteristics include magnetic properties (useful in magnetic separation, data storage, and magnetic resonance imaging (MRI)), high surface area (so they are highly reactive and useful for catalytic applications), biocompatibility (useful for biomedical applications, drug delivery, and imaging) [19]. However, there are several drawbacks associated with synthesis of iron nanoparticles, including agglomeration (Fe nanoparticles tend to agglomerate or clump together, which can reduce their effectiveness in certain applications), oxidation (they can easily oxidize when exposed to air or moisture, which can alter their properties and reduce their stability), etc. [20]. Nonetheless, the method of ‘green synthesis’ of Fe nanoparticles is advantageous, as it avoids the utilization of toxic chemicals and solvents. Additionally, it is cost-effective and can be scaled up for large-scale production [21]. The increased biological oxygen demand (BOD) and chemical oxygen demand (COD) levels are detrimental to the aquatic ecology. This situation causes dissolved oxygen (DO) levels to drop, which in turn creates anaerobic conditions that are harmful to aquatic species. Additionally, a higher BOD level in water indicates a high level of nutrients, which may cause an algal bloom. Dlamini et al. [22] reported on FeNPs with the best removal efficiency for impurities such as COD and BOD with removal efficiencies of 76% and 80%, respectively. There is less literature about the utilization of FeNPs synthesized from bioflocculant. To the best of our knowledge, a bioflocculant produced from Pichia kudriavzevii a yeast from Kombucha tea SCOBY has never been used to synthesize Fe nanoparticles and used for application in wastewater treatment.

This study describes a straightforward and environmentally sustainable approach in producing Fe nanoparticles. It involves utilizing a bioflocculant derived from *Pichia kudraivzevii* isolated from Kombucha tea SCOBY (symbiotic culture of bacteria and yeast), as a reducing and stabilizing agent. The resulting nanoparticles can then be used for treating wastewater, coal-mine water, and eliminating dyes. In addition, this research presents the assessment of the toxicity of Fe nanoparticles on human embryonic kidney (HEK 293) cells to see if the material can create any complications for human kidneys, since these nanoparticles will be applied to treat water that will eventually be used by humans. Finally, the use of Fe nanoparticles was optimized for the purpose of flocculation, and their potential antimicrobial properties were tested against various microorganisms belonging to Gram-negative and Gram-positive categories.

## 2. Results and Discussion

Supplementary data contain the characterization results of UV-vis spectroscopy, XRD, TGA, and TEM information regarding the synthesized Fe nanoparticles and the bioflocculant. Figure S1 (Supplementary Materials) illustrates the UV-vis spectra of both a bioflocculant and the biosynthesized Fe nanoparticles. Figure S1a displays the absorption peak at 230 nm observed in the bioflocculant. It has been reported that the presence of biomolecules from the bioflocculant, responsible for nanoparticle production, results in absorption peaks ranging from 200 to 340 nm [23]. The biosynthesized Fe nanoparticles showed surface plasmon resonance at 200 to 350 nm, with peaks observed between 210, 265 and 330 nm. The Fe nanoparticles were observed to have a close-to-spherical shape, as revealed by the TEM micrographs shown in Figure S2 (Supplementary Materials). The transmission electron microscope analysis determined that the size of the Fe nanoparticles ranged between 16.2 and 18.55 nm. Yadev et al. [24] reported similar findings, describing the spherical morphology of Fe nanoparticles derived from Aloe vera. X-ray diffraction is a powerful technique that effectively reveals the presence of crystallite and amorphous regions within the particles. Figure S3 (Supplementary materials) depicts the XRD pattern for both the bioflocculant and the Fe nanoparticles. The bioflocculant exhibited narrow and distinct diffraction peaks at 2θ = 20°, 23°, 25°, 30°, and 34°, indicating its crystalline nature. The as-synthesized Fe nanoparticles displayed broad peaks in the XRD pattern, observed at approximately 2θ values of 17.17°, 32.58°, 33.75°, 38.18°, 45.31°, 57.40° and 72.4°. The TG pattern for both the bioflocculant and FeNPs is depicted in Figure S4 (Supplementary materials). Both samples exhibit a three-phase disintegration pattern, as revealed by the thermogravimetric pattern. The biosynthesized FeNPs displayed a weight loss (2.8%wt) during the initial stage, which took place approximately between 50 to 100 °C. In contrast, the bioflocculant exhibited a weight loss (20.5%wt) within the temperature range of 50 to 150 °C. The temperature range of 150–210 °C for FeNPs and 150–500 °C for the bioflocculant can be attributed to the degradation of the polymer, second stage. For FeNPs, the third stage is observed within the temperature range of approximately 200 to 900 °C, while for the bioflocculant, it occurs between 500 and 900 °C.

### 2.1. Analysis Using Scanning Electron Microscopy (SEM)

**Figure 1:**
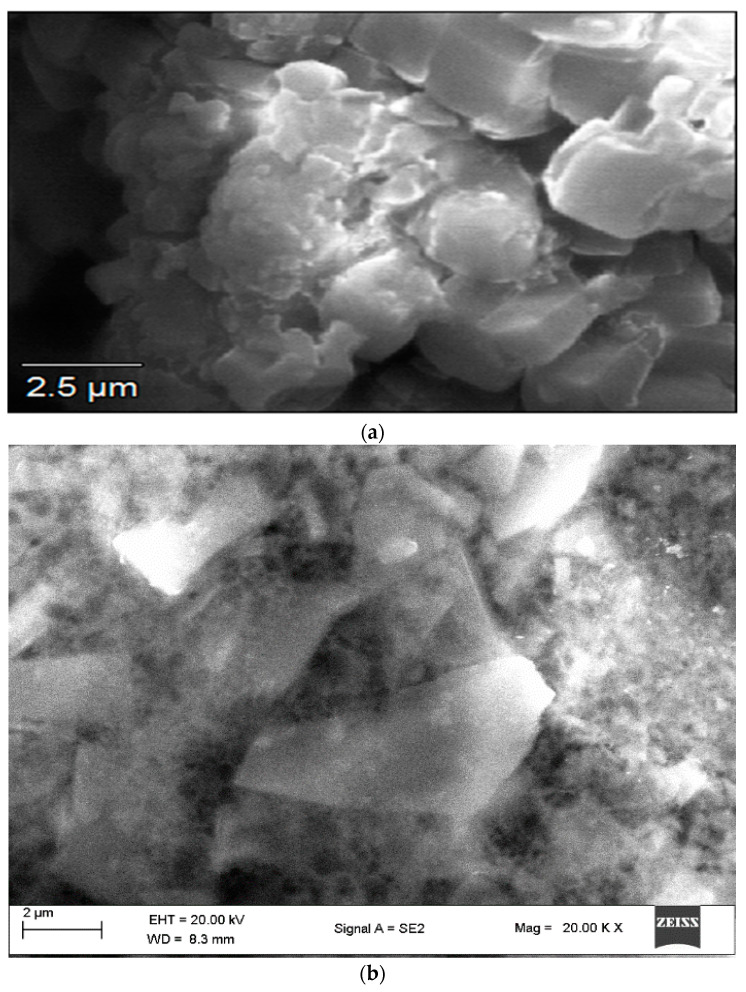
SEM micrographs of (**a**) bioflocculant and (**b**) as-produced Fe nanoparticles.

The SEM analysis aids in supplying details regarding the surface characteristics of the specimens. SEM imaging of Fe nanoparticles reveals their surface topography and enables the evaluation of their size and shape. Figure 1a shows a cumulus-like morphological structure of the bioflocculant. The shape of the FeNPs in their initial state appeared to be a distorted hexagonal structure, and they were found to be agglomerated, possibly as a result of the presence of the bioflocculant utilized during their synthesis (Figure 1b). The size and morphology of FeNPs vary depending on their type [25]. In another study, SEM examination of iron nanoparticles reported that the size fell within the range of 58 to 79 nm, and their morphology was observed to be spherical [26].

### 2.2. EDX Analysis

**Figure 2:**
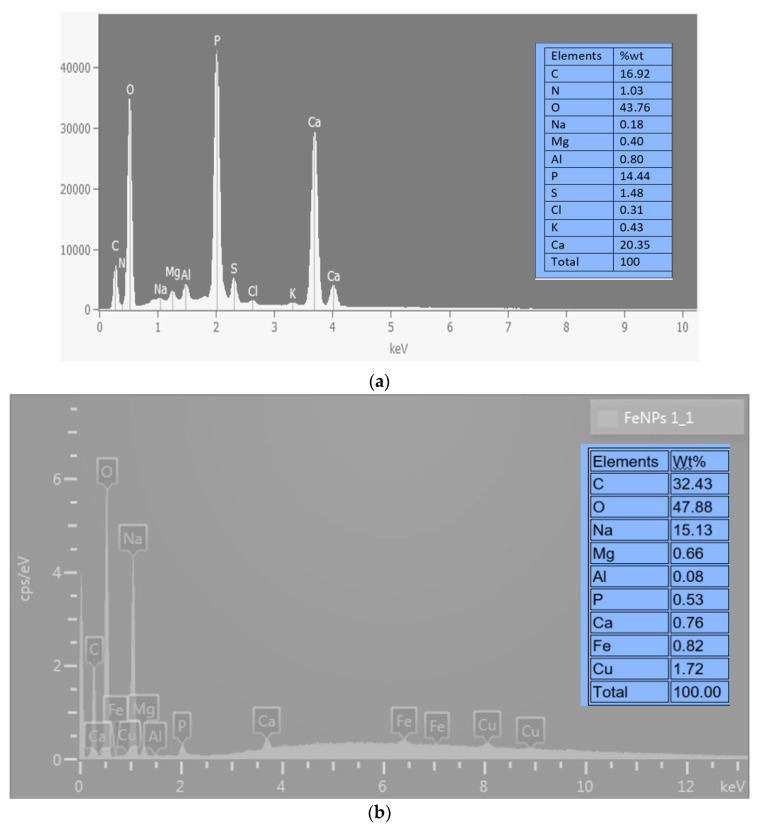
EDX analysis of (**a**) bioflocculant and (**b**) as-produced Fe nanoparticles.

Figure 2’s SEM-EDX analysis findings show that the bioflocculant and iron nanoparticles as prepared contain a variety of components. The bioflocculant (Figure 2a) is shown as having components like C, N, O, Na, Mg, Al, P, S, Cl, K, and Ca. O had the greatest weight percentage (wt%) at 43.76%, whereas Na had the lowest (wt%) at 0.18%. The bioflocculant-passivated iron nanoparticles (Figure 2b) show elements including C, O, Na, Mg, Al, P, Ca, Fe, and Cu. From this figure, it is clear that O was the dominating component, just as was observed from Figure 2a of the bioflocculant. The element oxygen had a weight percentage (wt%) at 47.88% and the least wt% was Al with just 0.08%. The presence of Fe with 0.82 wt% (Figure 2b) was an indication that iron nanoparticles were synthesized in the experiment, as this element (Fe) was not present from the EDX spectrum of the bioflocculant from which it was synthesized. The presence of impurities or contamination in the sample, which may be concealing the Fe signal, or inadequate dispersion and aggregation of the FeNPs, could account for the relatively low weight percentage of Fe [27]. The existence of oxygen and carbon within the specimen suggests that the bioflocculant utilized for synthesis is a type of a polysaccharide [22].

### 2.3. Functional Group Analysis

**Figure 3:**
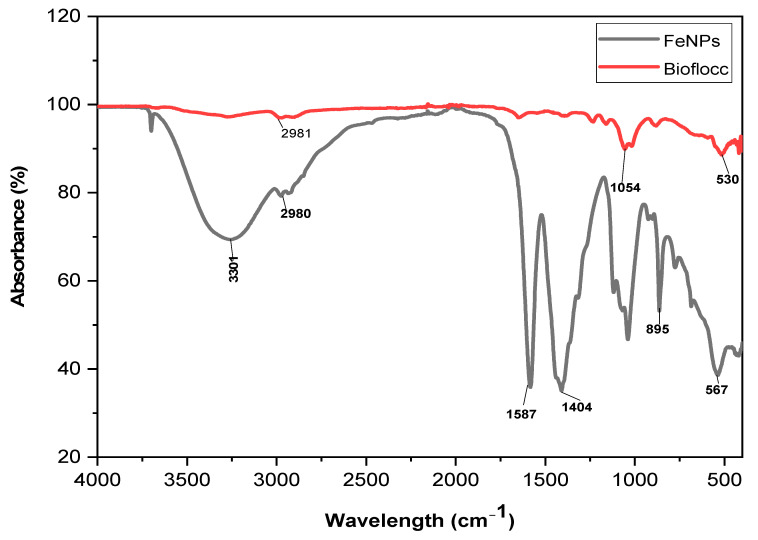
FT-IR spectra of a bioflocculant and as-prepared Fe nanoparticles.

The bioflocculant and the bioflocculant passivated FeNPs’ FT-IR spectra are shown in Figure 3. The peak observed at 3244 cm^−1^ in the sample (Fe nanoparticles) corresponds to the existence of both hydroxyl (–OH) and amine (–NH_2_) groups. These functional groups are responsible for producing Fe nanoparticles. The faint peak revealed at 1587 cm^−1^ can be explained by the existence of aliphatic bonds. The presence of an amide group is indicated by the peak detected at 978 cm^−1^. The presence of the –OH group is confirmed by the vibrational peaks observed at 567 cm^−1^, which is consistent with the C–O stretching commonly found in alcohols. The distinguishing feature of the alkane group is the presence of a C-H bond that exhibits a band value of 2981 cm^−1^.

The various functional groups revealed by Fourier-transform infrared spectroscopy (FT-IR) have been reported to be the key elements to the generation of nanoparticles. The literature has reported that biological substances like plant extracts and bioflocculants have functional molecules that are capable of reducing metal salts to create nanomaterials [28]. In addition, functional groups serve as attachment points for individual particles to come together and form larger clumps, a process known as flocculation [29].

### 2.4. Dosage Determination

**Figure 4:**
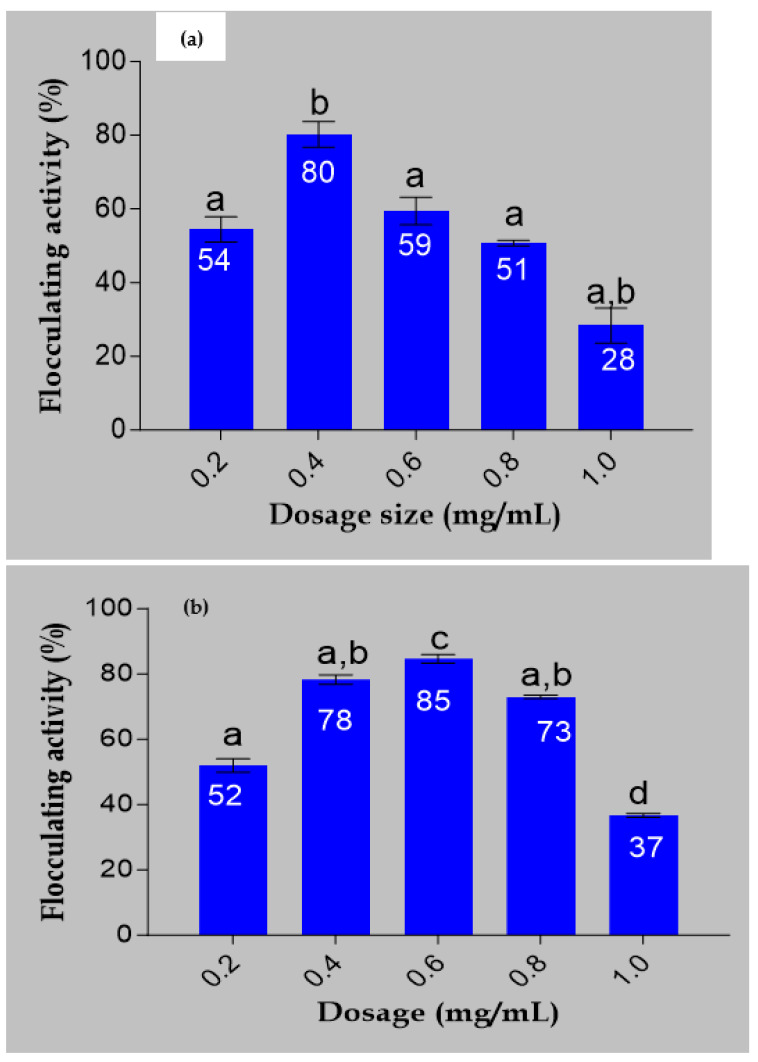
Dosage effect of the (**a**) bioflocculant and (**b**) Fe nanoparticles on flocculating activity. The letters a, b, c, and d represent statistical significance.

The flocculating activities of the bioflocculant and iron nanoparticles are demonstrated in Figure 4. The bioflocculat showed flocculating activity of 80% at a dosage size of 0.4 mg/mL. It can be shown that 0.6 mg/mL, which produced 85% flocculating activity, was the most efficient dosage for Fe nanoparticles (Figure 4b). These results reveal that when the bioflocculant is used to synthesize the Fe nanoparticles, the flocculating activity increases with an increase in dosage size until a certain point where there is drastic decline in flocculating activity. A study by Masuku [30] reported that, when the dosage concentration is high, it leads to the generation of high viscosity, which hampers the settling rate of solid particles in the solution. The present investigation yielded a comparable finding. Figure 4 indicates that the ideal flocculation rate of 85% was achieved at a dosage of 0.6 mg/mL. However, with an increase in concentration, a modest reduction in flocculating activity was noted. Dlamini et al. [22] reported FeNPs synthesized from a bioflocculant produced by Alcaligenes faecalis with 0.4 mg/mL optimum dosage concentration. FeNPs’ decreased ability to flocculate may have decreased due to the creation of high viscosity in the solution as a result of their high concentration. This high viscosity could potentially obstruct the site of adsorption for molecules and impede the movement of FeNPs required for the flocculation in the solution of suspended particles [31].

### 2.5. Antibacterial Activity Test

The MIC and MBC findings of the produced Fe nanoparticles, bioflocculant, and the common antibiotic Ciprofloxacin are shown in Table 1 for comparison. The biosynthesized Fe nanoparticles exhibited significant properties against both Gram-positive (Staphylococcus aureus and Bacillus cereus) and Gram-negative (Escherichia coli and Pseudomonas aeruginosa) microorganisms, with the least observed MIC and MBC against Bacillus cereus (3.125 and 6.25 mg/mL, respectively). Fe nanoparticles’ antibacterial effectiveness was contrasted with that of Ciprofloxacin, the experiment’s control drug. Furthermore, an amount of 20 µL of Ciprofloxacin was used, which exhibited inhibitory effects on all tested organisms. The Fe nanoparticles demonstrated significant properties of inhibiting both Gram-positive and Gram-negative microorganisms when compared to the bioflocculant used for their synthesis, which showed no antimicrobial effects on any of the tested organisms. When the MIC and MBC values are low, it indicates that a lower amount of drug is needed to halt the growth of the microorganisms. Antimicrobial drugs are thought to be more effective when the MIC and MBC values of the drug are low [32]. The study suggests that the Fe nanoparticles synthesized in this research could serve as an effective antibacterial agent in a variety of sectors, such as treatment of wastewater and production of food. Siddiqi and Husen [33] suggest that the reduced size of nanomaterials and metal ions compared to bacterial cells makes it probable for them to interfere with the cell membrane and impede bacterial cell growth. The study’s findings concur with those that have been reported by Vasantharaj et al. [34] where a FeNP synthesized from Ruellia tuberosa observed higher antimicrobial activity against E. coli and lesser activity against S. aureus. However, the bioflocculant showed no effect on the microbial strains tested in this experiment.

### 2.6. Cytotoxicity Analysis of FeNPs in Comparison to the Bioflocculant

**Figure 5:**
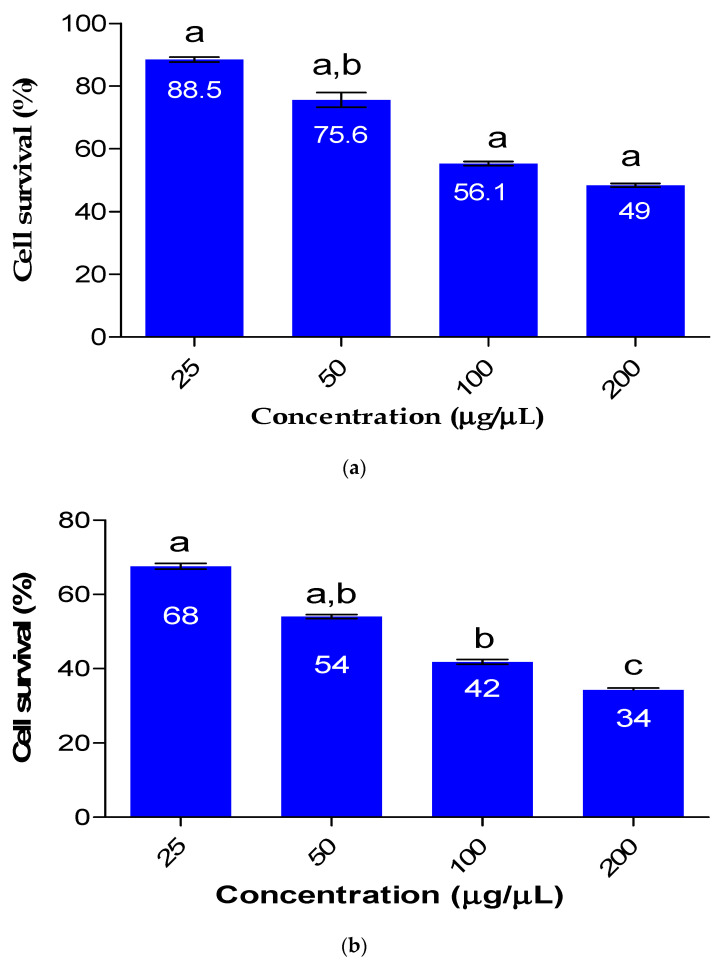
Biosafety profile of the bioflocculant (**a**) and as-produced Fe nanoparticles (**b**). The letters a, b, and c represent statistical significance.

The impact of Fe nanoparticles on HEK 293 cells is depicted in Figure 5. The viability of HEK 293 cells was concentration-dependent and was affected by the cytotoxicity of the synthesized Fe nanoparticles. As the use of nanomaterials continues to increase and their release into the environment becomes more prevalent, it is crucial to determine the toxicity of nanoparticles. When the Fe nanoparticles were used at a concentration of 25 µg/µL, cell survival was 68%, whereas at a much higher concentration of 200 µg/µL, only 34% of cell survival was observed. The bioflocculant used for the synthesis of Fe nanoparticles had a minimal effect on cell survival, with over 88.5% survival observed at a concentration of 25 µg/µL. The findings presented in the current study suggests that the Fe nanoparticles are safe to use in a low concentration. Dlamini et al. [22] reported Fe nanoparticle biosafety with cell survival of 56% for HEK 293 at the highest concentration of 100 µg/µL.

### 2.7. Color Removal

**Figure 6:**
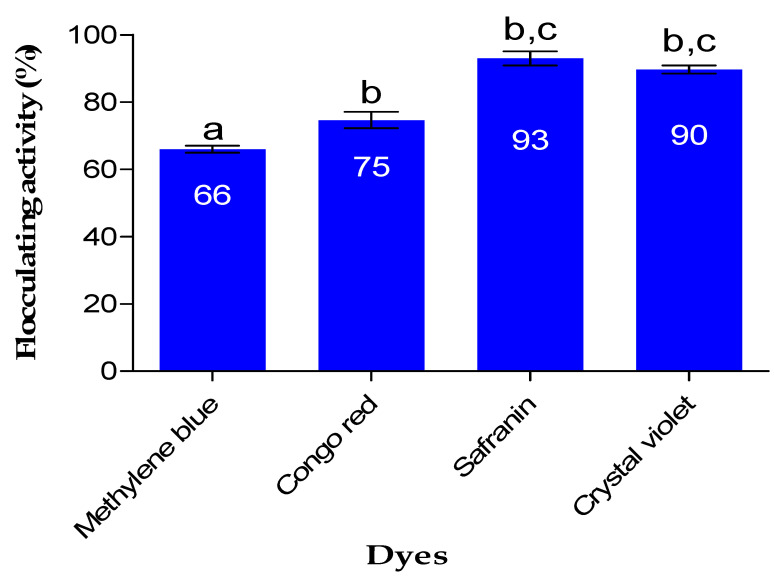
Removal efficiency of dyes from various solutions by as-produced Fe nanoparticles. The letters a, b, and c represent statistical significance.

The impact of Fe nanoparticles on the removal of dye is demonstrated in Figure 6. FeNPs demonstrated good removal efficiency of above 65% on all dyes investigated in the study. The aggregation of particles (Fe nanoparticles and dye particles) resulting in the removal of color from various solutions is primarily driven by the bridging and neutralization mechanisms [35]. The literature explains that the bridging mechanism occurs when flocculants affect the colloidal particle aggregation, extending from the surfaces of the particles into the suspension over a distance that exceeds the range of the inter-particle repulsion forces [36]. As a result of the bridging mechanism, the particles flocculate and form flocs. The as-prepared FeNPs removed safranin with 93% removal efficiency, which was the highest, while methylene blue was removed with 66% removal efficiency. In a study by Rather and Sundarapandian [37], it was reported that FeNPs synthesized by green method from a leaf extract of Wedelia urticifolia had a removal efficiency of 99% for methylene blue, while another study by Alshehri et al. [38] reported FeNPs synthesized from *Hibiscus sabdariffa* (Roselle) that showed a removal efficiency of 96.1% for Congo red.

### 2.8. Pollutant Removal from Wastewater

Table 2 illustrates the results of the evaluation of the efficacy of Fe nanoparticles produced via biological means in causing the aggregation of various colloidal and suspended particles present in wastewater from a coal mine (Tendele Coal mine, Somkhele area, Mtubatuba, KwaZulu-Natal, South Africa). The capacity of Fe nanoparticles to cause aggregation of suspended and colloidal particles was compared to a bioflocculant and one commonly used commercial flocculant (iron(III) chloride) in wastewater treatment.

Table 2 presents the results, indicating that iron nanoparticles have significant potential in the wastewater treatment from Tendele coal-mine industry, as they were able to effectively eliminate high proportions of COD (77%), BOD (87%), phosphorus (85%), sulfate (82%), and total nitrogen (73%), in addition to exhibiting high flocculation efficiency (98%). The outcomes obtained (for FeNPs) are comparable to those achieved with types of flocculants (bioflocculant and FeCl_3_ (0.6 mg/mL)) that were utilized in the investigation. Fe nanoparticles obtained better removal rates for COD, BOD, phosphorus, total nitrogen, and sulfate when compared to the bioflocculant and commercial flocculant FeCl_3_. Based on the data presented in Table 2, it can be inferred that Fe nanoparticles have the potential to serve as viable substitutes for conventional flocculants. Moreover, unlike conventional flocculants, Fe nanoparticles synthesized using flocculants possess traits such as biodegradability and eco-friendliness. The ability of Fe nanoparticles to address COD and BOD in wastewater from a coal mine more effectively than chemical flocculants is particularly intriguing, as elevated levels of these substances in water can have adverse impacts on aquatic organisms [39]. The capacity of Fe nanoparticles to remove contaminants appears to be primarily influenced by their chemical components, surface structure, and functional groups. The findings of these investigations suggest that Fe nanoparticles hold significant promise for use in various industrial applications, especially in wastewater treatment industries. Furthermore, the fact that Fe nanoparticles can eliminate various types of chemicals from wastewater indicates their versatility.

## 3. Materials and Methods

### 3.1. Bioflocculant Production and Purification

The procedure for extracting and purifying the bioflocculant from *Pichia kudriavzevii*, as described by Tsilo et al. [40], was employed with slight alterations. A mixture of fermentation was created by combining 10 g of peptone, 0.1 g of (NH_4_)_2_SO_4_, 1.0 g of KH_2_PO_4_, 2.5 g of K_2_HPO_4_, 0.05 g of NaCl, and 0.1 g of MgSO_4_ with 500 mL of Kombucha tea broth. Prior to sterilization in an autoclave at 121 °C for 15 min and inoculation with 2% (*v*/*v*) culture broth, the medium was adjusted to a pH of 7. The fermentation mixture that was infused with the culture was kept in an incubator at 35 °C and agitated at 140 revolutions per minute for a duration of 60 h. The fermented broth culture underwent centrifugation at 8000× *g* at 4 °C for 15 min to isolate bacterial cells. The bacterial cells were purified by mixing the supernatant with 1 liter of distilled water, followed by second round of centrifugation at 8000× *g* for 15 min at a temperature of 4 °C. The supernatant received an addition of two liters of ice-cold ethanol. The mixture was agitated vigorously, following which it was allowed to settle for 12 h at a temperature of 4 °C to induce precipitation. Subsequently, the precipitate was subjected to vacuum drying to yield the unrefined bioflocculant. The unrefined bioflocculant was dissolved in 100 mL of distilled water to create a solution with a certain weight-to-volume ratio (*w*/*v*). A 5:2 mixture of chloroform and n-butyl alcohol was added, thoroughly mixed, and left to stand at room temperature for a duration of 24 h. The bioflocculant in its pure form was acquired by centrifugating the supernatant at 8000× *g* for 15 min at a temperature of 4 °C and then drying under a vacuum.

### 3.2. Iron Nanoparticles (FeNPs) Biosynthesis

A method utilizing green techniques was employed to generate iron nanoparticles from iron sulfate (FeSO_4_) as a metal precursor [41]. In order to prevent nanoparticle agglomeration, 10 mL of 5.0 M sodium hydroxide (NaOH) solution was added. In total, 0.5 g of pure bioflocculant was dissolved in 10 mL of 0.2 M iron sulfate (FeSO_4_) solution. The blend was allowed to sit at standard temperature for 24 h, and the generation of nanoparticles was verified through physical examination such as the change in color and analysis. Afterwards, the mixture was spun at 5000 rotations per minute at a temperature of 4 °C for a duration of 15 min, in order to obtain the produced nanoparticles. The resulting sediment was then subjected to a vacuum drying process at a temperature of 25 °C for 24 h, as stated in a method by de Jabber et al. [42].

### 3.3. Characterization of the Purified Bioflocculant and Biosynthesized FeNPs

To examine the elemental composition and physical structure of both the bioflocculant and Fe nanoparticles, energy-dispersive X-ray spectroscopy (EDX) and scanning electron microscope (SEM) analyses were conducted using equipment from JEOL, USA, Inc., based in Peabody, MA, USA. Additionally, the JEOL 1010 transmission electron microscope (JEOL USA, Inc., Peabody, MA, USA) was utilized to capture the TEM micrographs.

The bioflocculant and Fe nanoparticle functional groups were identified and confirmed using a Fourier-transform infrared (FT-IR) spectrophotometer (Bruker’s Tensor 27 model. Gauteng, South Africa). The used parameters have a resolution of 4 cm^−1^ and a range of 4000 to 400 cm^−1^.

Optically measuring the produced Fe nanoparticles was done with the Varian Cary 50 Conc UV-vis spectrophotometer (PerkinElmer, Gauteng, South Africa). Using diluted samples, the wavelength spanned from 200 to 700 nm, and the resolution was 1 nm. We utilized the Cu-Kα radiation (λ = 1.5406 Å)-equipped Bruker Advance D8 diffractometer (Bruker, Gauteng, South Africa) to record the diffraction patterns of the freshly produced Fe nanoparticles.

The thermogravimetric analysis of the synthesized FeNPs was performed using the PerkinElmer Thermal Analysis Pyris 6 TGA (PerkinElmer, Inc., Waltham, MA, USA). The degradation patterns of the samples were obtained by subjecting them to a nitrogen gas flow rate of 40 cc/min and a temperature range of 22 to 900 °C, with a temperature ramping rate of 10 °C/min.

### 3.4. Determination of Dosage Concentration

Iron nanoparticles were diluted with distilled water to create solutions with 0.2, 0.4, 0.6, 0.8, and 1.0 mg/mL concentrations. Each solution was prepared separately by diluting the Fe nanoparticles to the desired concentration. The test material employed was kaolin clay. A liter of distilled water was mixed with four grams of kaolin clay to make a solution. Then, 100 mL of this kaolin solution was mixed with 3 mL 1% (*w*/*v*) CaCl_2_ and 2 mL of Fe nanoparticles in a 250 mL conical flask [43]. After being vigorously mixed for one minute, the mixture was then transferred into a measuring cylinder (100 mL) and left undisturbed for five minutes at a temperature of 25 °C, during which time sedimentation occurred. The same process was repeated for the control, except that 2 mL de-ionized water was used instead of the 2 mL of Fe nanoparticles. The transparent upper layer of the supernatant was extracted using a pipette and placed into a cuvette to assess the flocculation performance [44]. The flocculation rate of both the Fe nanoparticles and the control was determined by measuring the optical density (OD550nm) of the liquid utilizing a UV-visible spectroscopy (PerkinElmer, Gauteng, South Africa), which was positioned 1 cm below the fluid surface. The measurements were based on the results obtained from the spectrophotometer. The below equation was used to compute the flocculation activity (FA):

FA (%) = [ (A − B)/A]100(1)

In the equation, A refers to the optical density at 550 nm of the untreated control and B represents the Fe nanoparticle-treated suspension.

### 3.5. Antibacterial Assay

#### 3.5.1. Revival of Microbial Strains

The microbial strains (Gram-positive and Gram-negative) were placed on individual sterilized nutrient broths and subsequently incubated for 24 h at 37 °C. In separate test tubes, 1 mL of *Staphylococcus aureus*, *Bacillus cereus*, *Pseudomonas aeruginosa*, and *Escherichia coli* ATCC 25922 strains were each added in 9 mL of sterile nutrient broth. The blend was kept in a 37 °C incubator for a duration of 12 h. The bacterial absorbance/turbidity was assessed at 600 nm using a UV-visible spectrophotometer, and then adjusted to match the McFarland standards (0.5) [45].

#### 3.5.2. Minimum Inhibitory Concentration and Minimum Bactericidal Concentration

To prepare the nutrient broth, 23 g of the substance were suspended in 1 liter of distilled water, and the consequential solution was sterilized by autoclaving at 121 °C for 15 min. A positive control was established using 40 μL of Ciprofloxacin. A set of dilution factors were created using a 96-well plate. In all wells of the micro-plate, 50 μL of sterile nutrient broth was first added, and then, 50 µL of bacterial isolate was added to each adjacent well in a descending order of concentration. In each respective well, 50 μL of FeNPs was added, and subsequently, the well containing distilled water was utilized as the negative control, and 50 µL Ciprofloxacin was inoculated to it. Following overnight incubation at 37 °C, the addition of 40 μL of a 0.2 mg/mL p-iodonitrotetrazolium (INT) indicator solution led to the observation of color changes in the solutions [46].

The plates were assessed for minimum bactericidal concentration, which demonstrated a positive minimum inhibitory concentration. Cultures from each strain were streaked onto Mueller Hilton nutrient agar using a loop filled with culture, and no color change was observed. Then they were incubated for 12 h at 37 °C. The MBC was established as the lowest amount of Fe nanoparticles necessary to completely eradicate the test organisms.

### 3.6. Cytotoxicity Assay

The technique outlined by Bhatia and Bhatia [47] was utilized to assess the cytotoxicity effects of the particles (bioflocculants and Fe nanoparticles) on a human embryonic kidney (HEK 293) cell line. Cell suspensions containing 1 × 10^5^ cells/mL of dosage concentrations were seeded onto a 96-well micro-plate. The cells were seeded with varying concentrations of Fe nanoparticles (ranging from 25 to 100 µg/µL) using a method involving tenfold serial dilution. Following a 48-h incubation period, Fe nanoparticles were administered using media containing 1% fetal bovine serum (FBS), and subsequently, the plates were placed back in the incubator for another 48 h. Each of the wells was then filled with 15 µL of MTT solution (5 mg/mL) in phosphate-buffered saline (PBS), which was then incubated for 4 h at 37 °C. The MTT-containing media and the resulting formazan crystals were removed from the wells and then dissolved in 100 mL of dimethyl sulfoxide (DMSO). The optical densities of the solutions at a wavelength of 570 nm were measured using a micro-plate reader. The following equation was used to compute the percentage of cell inhibition:

Cell viability (%) = [C_i_/C_f_] × 100 (2)

In the equation, C_f_ represents the final value recorded after the treatment with the flocculant, while C_i_ signifies the initial value obtained prior to the treatment.

### 3.7. Efficiency of Dye Removal Using Fe Nanoparticles

An experiment was performed to determine the removal efficiency (RE) of the dyes. This entailed mixing 100 mL of solution containing each dye at a concentration of 4 g/L with 2 mL of iron nanoparticle solution (0.6 mg/mL) and 3 mL of 1% *w*/*v* CaCl_2_; the pH of the solution was pH 6. The dyes used in the experiment were safranin (OD_550nm_), methylene blue (OD_660nm_), Congo red (OD_500nm_), and crystal violet (OD_590nm_), and they were measured at their respective optical densities (OD). After leaving the mixture for a 10-min period at room temperature, the top face was gathered for examination at each dye’s maximal wavelength using a cuvette. The below equation provided was utilized to calculate the removal efficiency (RE):

RE (%) = [(C_0_ − C_1_)/C_0_] × 100 (3)

The variables used in the equation are as follows: C_0_ represents the initial value and C_1_ represents the value following flocculation treatments [48].

### 3.8. Wastewater Treatment

The effectiveness of the flocculant (FeNPs) in removing the coal mine effluent from Tendele Coal Mine, Mtubatuba, KwaZulu-Natal, RSA, was investigated. To assess biological oxygen demand (BOD), chemical oxygen demand (COD), total nitrogen (N), phosphate (PO_4_^3−^), and sulfate (SO_4_^2−^), removal, the approach outlined by Dlamini et al. [49] was utilized. Before the treatment, using either 1 M NaOH and 1 M HCL, the samples were treated to bring their pH level to 6. In order to carry out the Jar test, 100 mL of wastewater sample was combined with 3 mL of 1% (*w*/*v*) Ca2+ solution and 2 mL of a solution containing Fe nanoparticles (0.2 mg/mL), and the pH was adjusted to 6. Some of the samples were processed using bioflocculant solution with concentration of 0.2 mg/mL, while others were subjected to treatment with conventional flocculant like polyacrylamide, which was employed as a benchmark. The mixtures were agitated at room temperature, with an initial shaking at 200 rpm for a duration of 3 min, which was subsequently lowered to 45 rpm for 5 min. Afterwards, at the ambient temperature, the flasks were allowed to settle for a period of 10 min prior to conducting the analysis. For the purpose of determining removal efficiency (RE), a Pharo 300 Spectroquant^®^ UV-Vis spectrophotometer (Merck, Gauteng, South Africa) was employed and set to a wavelength of 680 nm. The Equation (3) above was utilized to compute the removal efficiency of pollutants from wastewater.

### 3.9. Statistical Evaluation

The data used for the experiment were gathered in triplicate, and the error bars displaying the data’s standard deviation are shown in the figures. GraphPad Prism version 6.1 was used to perform a One-Way Variance analysis on all the obtained data, with a significance threshold of *p* < 0.05 being used to determine statistical significance.

## 4. Conclusions

The bioflocculant was used to produce the Fe nanoparticles, which were then characterized using the SEM-EDX and FT-IR techniques. The biosynthesized Fe nanoparticles showed optimum dosage at a concentration of 0.6 mg/mL with flocculating activity of 85% against a kaolin solution. The antimicrobial properties of Fe nanoparticles were significant, as all the Gram-negative and Gram-positive pathogenic bacteria tested showed favorable results. The nanoparticles exhibited notable cytotoxic effects on HEK 293 cell lines in a manner that is dependent on the concentration. Compared to conventional flocculants such as iron (III) chloride, they show a greater potential for eliminating various pollutants in coal-mine wastewater. FeNPs displayed an exceptional removal efficiency for both COD and BOD. The Fe nanoparticles can eliminate more than 65% of the dyes present in various dye solutions, with the maximum removal efficiency of 93% achieved for Congo red solution using a 0.6 mg/mL dosage. Therefore, biosynthesized Fe nanoparticles have a significant potential to replace synthetic flocculants in wastewater treatment processes, especially industrial wastewater.

## Figures and Tables

**Table 1:** Comparing the MIC and MBC of Fe nanoparticles with the bioflocculant and Ciprofloxacin.

Bacteria	Fe Nanoparticles		Ciprofloxacin		Bioflocculant	
	MIC (mg/mL)	MBC (mg/mL)	MIC (mg/mL)	MBC (mg/mL)	MIC (mg/mL)	MBC (mg/mL)
*Staphylococcus aureus*	25.0	-	6.25	12.5	-	-
*Bacillus cereus*	3.125	6.25	1.56	3.125	-	-
*Escherichia coli*	12.5	25.0	3.125	6.25	-	-
*Pseudomonas aeruginosa*	6.25	12.50	6.25	6.25	-	-

**Table 2:** Potential of FeNPs to remove various pollutants from Tendele coal-mine wastewater in comparison to the bioflocculant and FeCl_3_.

Flocculants	Water Quality	COD (mg/L)	BOD (mg/L)	P (mg/L)	S (mg/L)	N (mg/L)	Flocculating Activity (%)
Microbial	Before treatment	146.6 ± 0.0	203 ± 0.1	14.5 ± 0.0	33.4 ± 0	9.0 ± 0.2	2.982
	After treatment	53.8 ± 0.0	55 ± 0.3	6.3 ± 1.0	11.3 ± 3.1	3.2 ± 0.3	0.275
	**Removal rate (%)**	**63**	**73**	**57**	**66**	**64**	**91**
FeNPs	Before treatment	146.6 ± 3.0	203 ± 0.0	14.5 ± 0.2	33.4 ± 0.1	9.0 ± 0.3	2.982
	After treatment	33.2 ± 0.2	25.4 ± 0.0	2.2 ± 0.2	6.1 ± 0.1	2.4 ± 0.0	0.053
	**Removal rate (%)**	**77**	**87**	**85**	**82**	**73**	**98**
FeCl_3_	Before treatment	146.6 ± 0.1	203 ± 0.0	14.5 ± 0.4	33.4 ± 0.0	9.0 ± 0.0	2.982
	After treatment	37.3 ± 0.2	52 ± 0.2	3.5 ± 0.0	7.4 ± 0.2	3.3 ± 1.0	0.293
	**Removal rate (%)**	**75**	**74**	**76**	**78**	**63**	**90**

## Data Availability

No data available.

## Supplementary Materials

The supporting information can be downloaded at: https://www.mdpi.com/article/10.3390/ijms241914731/s1.
