# Supplementary material for: Application of Iron Nanoparticles Synthesized from a Bioflocculant Produced by Yeast Strain Pichia kudriavzevii Obtained from Kombucha Tea SCOBY in the Treatment of Wastewater
Source: Int J Mol Sci. 2023 Sep 29;24(19):14731. doi: 10.3390/ijms241914731 (PMC10572716; doi:10.3390/ijms241914731)
Supplement: Supplementary file 1 [file ijms-24-14731-s001.zip › ijms-2607814-supplementary.pdf]

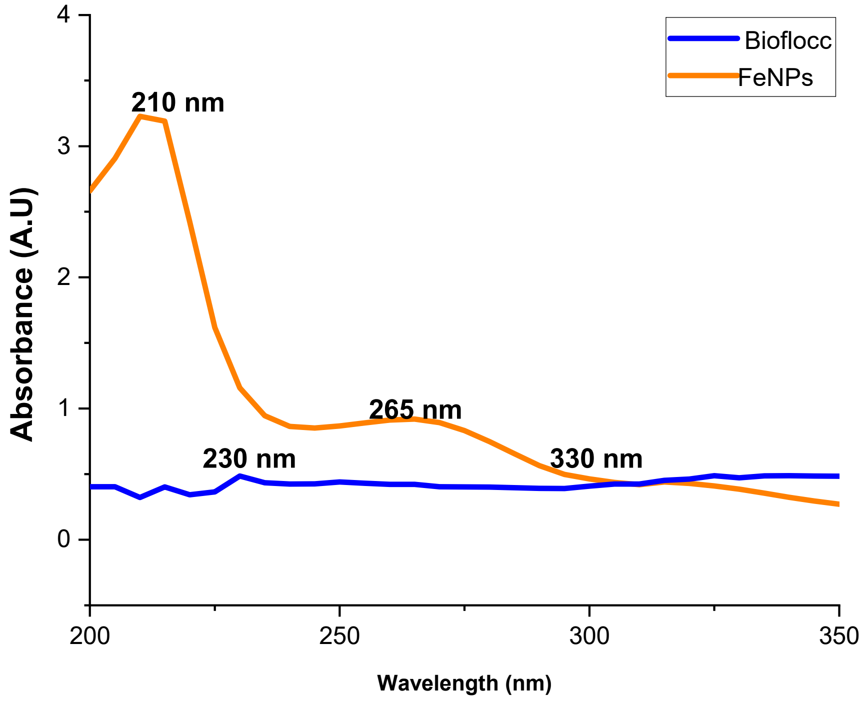

Figure S1. UV-Visible spectra of the bioflocculant and as-prepared Fe nanoparticles.

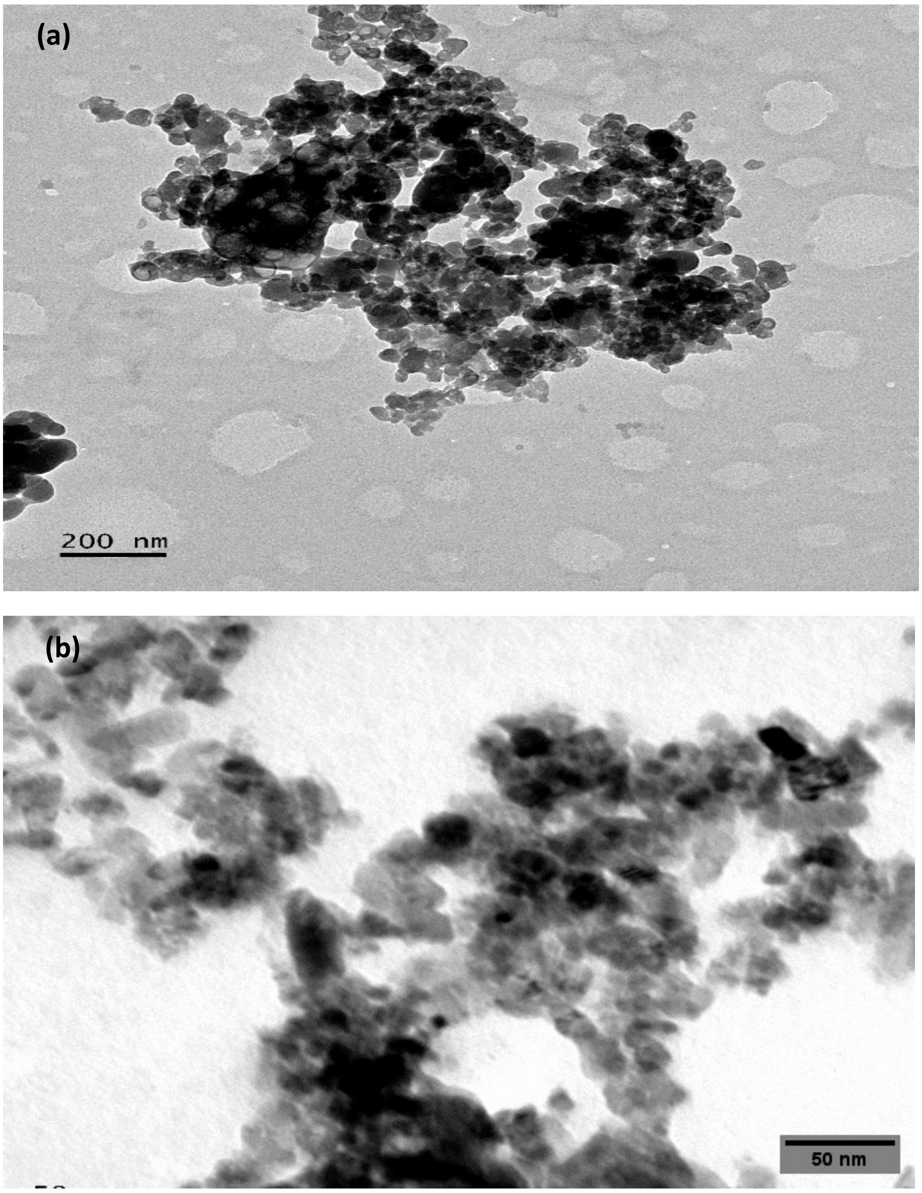

Figure S2. TEM micrographs of (a) bioflocculant and (b) as-prepared Fe nanoparticles.

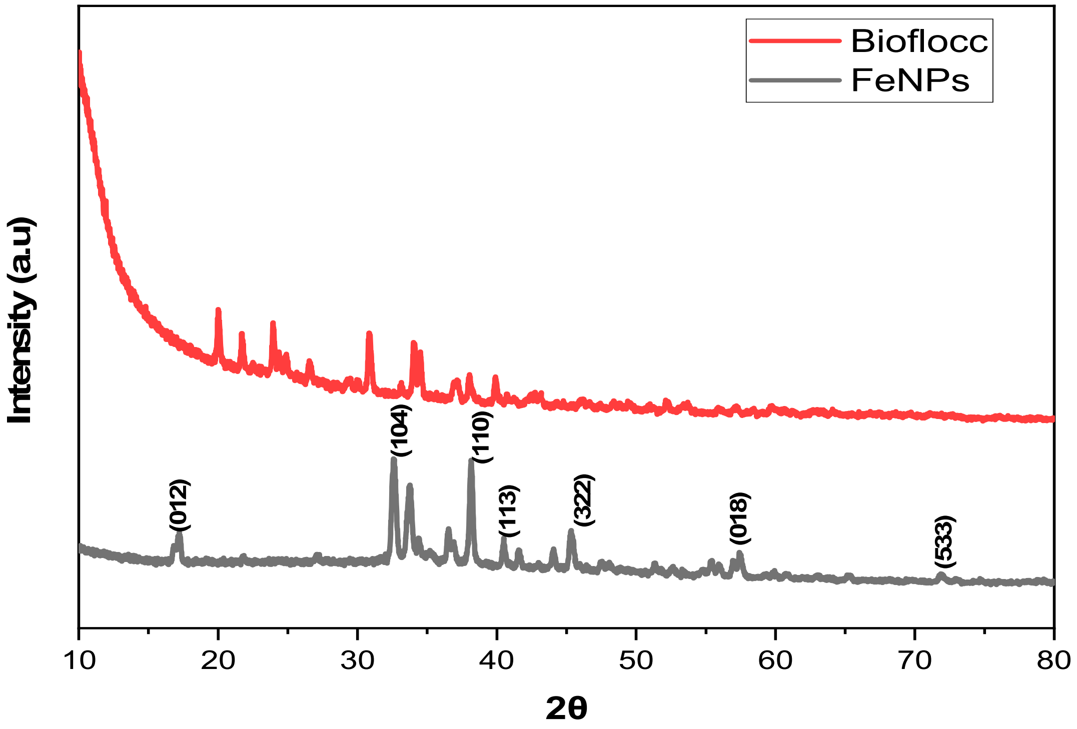

Figure S3. X-ray diffractogram of the bioflocculant and as-prepared Fe nanoparticles.

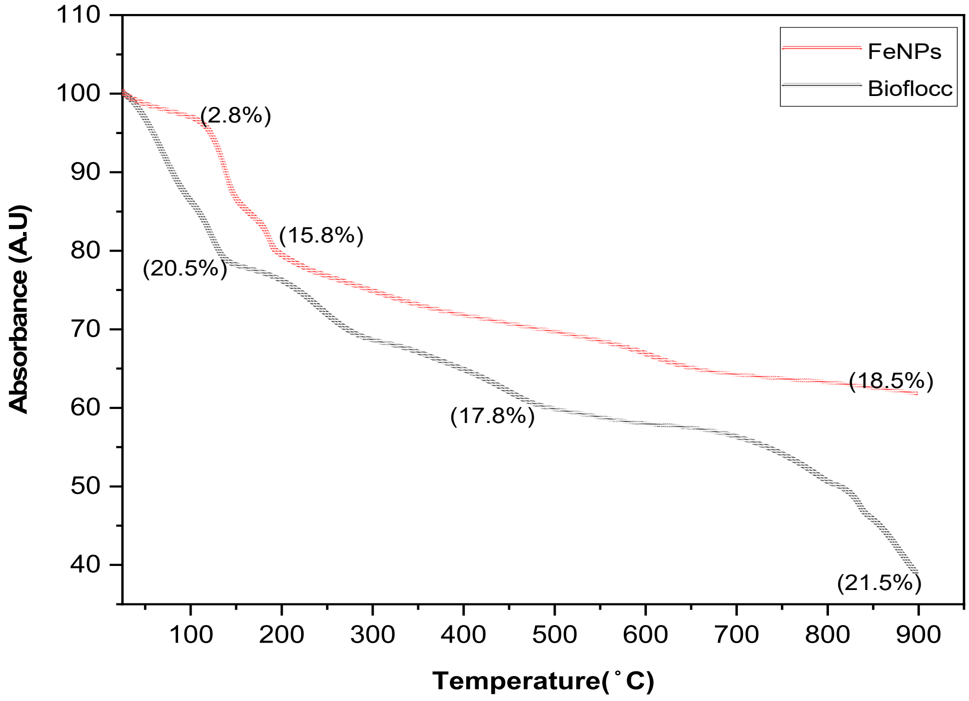

Figure S4. Thermogravimetric spectra of bioflocculant and Fe nanoparticles.
